# Development of optical microneedle–lens array for photodynamic therapy

**DOI:** 10.1007/s10544-025-00735-4

**Published:** 2025-01-28

**Authors:** Jongho Park, Jingzong Zhang, Beomjoon Kim

**Affiliations:** 1 Institute of Industrial Science, The University of Tokyo, Meguro-Ku, 153-8505 Tokyo Japan; 2 Department of Precision Engineering, School of Engineering, The University of Tokyo, Bunkyo City, 113-8656 Tokyo Japan

**Keywords:** Photodynamic therapy, Microneedle array patch, Optical microneedle–lens array, Non-melanoma skin cancer, TMPyP

## Abstract

Recently, photodynamic therapy (PDT) which involves a photosensitizer (PS), a special drug activated by light, and light irradiation has been widely used in treating various skin diseases such as port-wine stain as well as cancers such as melanoma and non-melanoma skin cancers. PDT comprises two general steps: the introduction of PS into the body or a specific spot to be treated, and the irradiation process using a light source with a specific wavelength to excite the PS. Although PDT is gaining great attention owing to its potential as a targeted approach in the treatment of skin cancers, several limitations still exist for practical use. One of the biggest challenges is the limited penetration of light owing to scattering, reflection, and absorption of light inside the skin layers. In addition, accidental light exposure of the target area causes additional cellular damage, which causes unexpected complications. To solve these issues, we introduced an optical microneedle–lens array (OMLA) to improve the efficiency and safety of PDT treatment. We designed and fabricated a novel optical microneedle–lens array with controlled dimensions to optimize light transmission. In addition, PS was coated uniformly over the tips of the OMLA using the dip coating method. Finally, we confirmed that the PS coated on the OMLA was released into the target area and subsequently generated radical oxygen by light irradiation. We expect that our proposed OMLA for PDT treatment can realize a new light-transmission platform optimized for PDT with targeting various types of skin cancers.

**Graphical abstract:**

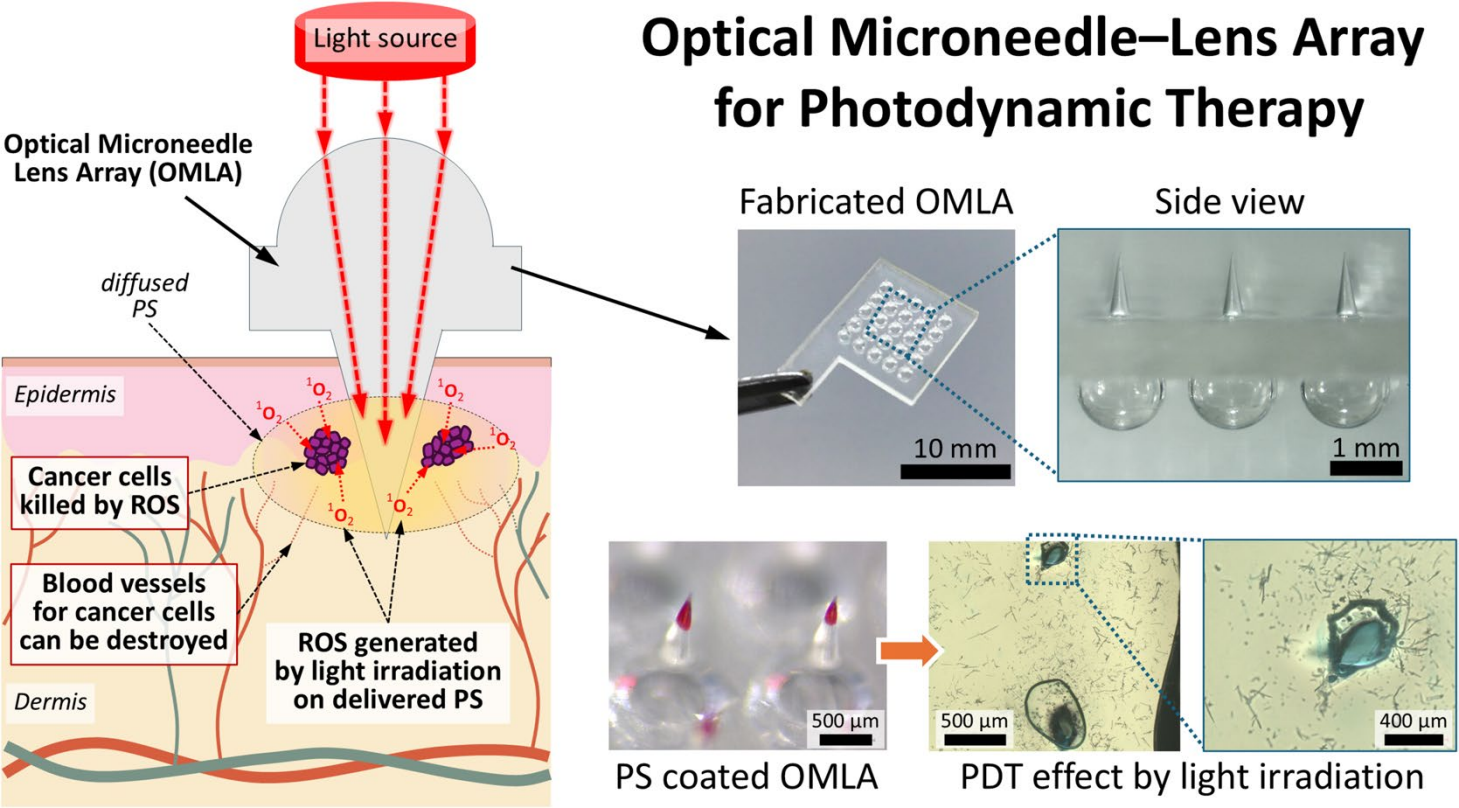

**Supplementary Information:**

The online version contains supplementary material available at 10.1007/s10544-025-00735-4.

## Introduction

Phototherapy is a medical treatment that uses light to treat various skin problems and diseases. This involves exposing the skin to specific wavelengths of light, which helps reduce inflammation, kill microorganisms, or promote wound healing. Modern phototherapy originated in the 19th century using ultraviolet (UV) irradiation and revealed its therapeutic possibilities in the dermatology field (Grzybowski et al. 2016). An important milestone was achieved when Niels Finsen received the Nobel Prize in 1903 for his pioneering work on treating lupus vulgaris using phototherapy (Grzybowski et al. 2012). While conventional phototherapy using natural sunlight has also been used for the treatment of tuberculosis and leg ulcers during wartime, modern phototherapy that uses artificial light sources has been gradually employed in dermatology to treat skin diseases and has become an effective and widely used treatment method.

Modern applications of phototherapy include various treatments that use different types of light to treat several skin diseases, such as psoriasis, vitiligo, eczema, and skin cancers. These treatments include laser therapy, low-level laser therapy (LLLT), intense pulsed light therapy (IPL), and photodynamic therapy (PDT). Laser therapy uses laser light created through a process called light amplification by the stimulated emission of radiation. It has several characteristics such as high coherence and directionality, linear polarization, and monochromaticity. The strength of the laser beam is attributed to these inherent characteristics, which make laser light an effective tool in the medical field. Thus, it was first implemented in the ophthalmology field for retinal surgery in the 1960s (Koutná et al. 2003). LLLT, also known as cold laser therapy, uses low-frequency continuous lasers to reduce pain and stimulate healing (Dima et al. 2017). Red and near-infrared lights with wavelengths from 600 to 1000 nm and output powers from 5 to 500 mW are being used clinically (Seifi et al. 2007). LLLT has been known for photo bio-stimulation and photo biomodulation, and other applications involving medical lasers or light-emitting diodes (LEDs). In addition, LLLT has been effectively deployed to treat a variety of situations, notably for its ability to induce biomodulation in cellular metabolism, along with providing analgesic and anti-inflammatory benefits.

In contrast to laser-based therapies, IPL use flash lamps and bandpass filters to release high-intensity, polychromatic light pulses with a defined wavelength spectrum, fluence, and pulse duration. Similar to lasers, the core mechanism of IPL causes selective thermal damage to a target (Babilas et al. 2010). Nevertheless, the use of specific wavelengths, pulse durations, and intervals makes it possible to treat a wide range of skin diseases. In addition, IPL has extended its application in cosmetic skin treatments, such as skin whitening (Kawada et al. 2008) and skin rejuvenation (Negishi et al. 2006).

Next, PDT is one of phototherapies, which is based on photodynamic reactions: the use of a photosensitizer combined with light of specific wavelength to destroy target cells. The toxic biochemical reaction is mediated by oxygen in PDT. The photosensitizer absorbs a light photon with a specific wavelength and transfers most of the absorbed energy to an oxygen molecule, which generates a relatively powerful oxidizing agent, named singlet oxygen (Darlenski and Fluhr 2012). Finally, light-induced singlet oxygen exerts cytotoxic effects by causing lethal oxidative damage to target cells in tissues where photosensitizers accumulate.

Among the various skin-related diseases, skin cancers have recently become an important target for phototherapy. Currently, there are two major types of skin cancer: melanoma and non-melanoma. Although non-melanoma skin cancer (NMSC) can be considered less aggressive and deadly than melanoma, it is the most common type, accounting for one-third of all malignancies worldwide (Leigh 2014). NMSC mainly includes basal cell carcinoma and squamous cell carcinoma, and the primary cause is the exposure of the skin to sunlight (Zambrano-Román et al. 2022). Thus, NMSC predominantly occurs in sun-exposed areas, mostly in the epidermal layer of the skin. Although it can be almost curable when diagnosed and treated at the earliest stage, the growth of skin cancer will gradually deepen without timely treatment, which will lead to aesthetic problems and even life-threatening conditions. Since 1990, the incidence and number of deaths from NMSC have been increasing globally at an annual rate of over 7% and 4%, respectively (Hu et al. 2022).

Basal cell carcinoma (BCC) typically manifests in sun-exposed areas of the body, with the highest incidence observed in the head and neck (80% of cases), followed by the trunk (15% of cases) and extremities. In addition, other sites where BCCs have been reported include the axillae, breasts, perianal area, genitalia, palms, and soles (Rubin and Chen 2005). Ultraviolet (UV) exposure is widely recognized as the predominant cause of BCC. Furthermore, certain factors such as fair skin, gender, age as well as immunosuppression have been identified as potential contributors to BCC development (Attal et al. 2024). Currently, the therapeutic options for BCC encompass both surgical and nonsurgical approaches. Surgical methods include the procedures like excision of cancerous tissue and cryosurgery, which generally exhibit high cure rates. Nonsurgical methods include radiation therapy, topical therapy, and photodynamic therapy. However, the tissue clearance rates achieved through nonsurgical approaches appear to be suboptimal, although photodynamic therapy does offer cosmetic benefits (Zhang et al. 2020).

Another type of NMSC is squamous cell carcinoma (SCC). SCC is the second most common type of skin cancer, typically arising from precancerous lesions, such as actinic keratosis and Bowen’s disease (Stratigos et al. 2015). It also occurs predominantly in sun-exposed areas of the body including the head, neck, and trunk. Similar to other skin cancers, prolonged exposure to UV radiation, such as UVA and UVB, has been identified as a primary risk factor for SCC (D’Orazio et al. 2013). In addition, circumstances such as chronic wounds, infections, and inflammatory skin diseases create an environment conducive to the genesis of malignant cells, which poses a significant challenge in the early identification of SCC, potentially causing a delay in diagnosis and treatment (Que et al. 2018). SCC has been treated using several methods, such as surgical and nonsurgical treatments. Although surgical excision remains the gold standard, nonsurgical treatments including PDT, laser ablation, and radiation therapy are also widely considered (Combalia and Carrera 2020).

Over the past several decades, the photodynamic effect has been the subject of extensive research and widely used in clinical treatment (Dolmans et al. 2003). The earliest PDT, light therapy using photodynamic reaction, dates to 1903 when Herman von Tappenier and A. Jesionek attempted to treat skin tumors with topically applied eosin and white light. A significant breakthrough was reported in 1960 by R.L. Lipson who showed the localized accumulation of the first practicable PDT drug, a photosensitizer (PS) known as hematoporphyrin derivative (HPD), to tumors and its fluorescence (Lipson et al. 1961). Since its first demonstration of PS, PDT has been widely researched and developed as a practical therapeutic application during the 1960s and the 1970s, including the eradication of mammary tumors in mice (Dougherty et al. 1975), elimination of bladder carcinoma (Kelly et al. 1975), and the first human trials with HPD (Kelly and Snell 1976). So far, various PS have been employed for PDT to treat skin cancers clinically. They include 5-Aminolevulinic acid (5-ALA), hematoporphyrin derivatives (HPD), meta-tetra(hydroxyphenyl) chlorin (mTHPC), and so on (Aziz et al. 2023).

PDT is based on the photochemical behavior of photosensitizers (PSs). The ground state of PS molecules, having two electrons with opposing spins in their low-energy molecular orbit, undergoes transformation into a short-lived excited singlet state upon light absorption. From this state, the excited PS can either return to its ground state through fluorescence emission or internal conversion, both of which involve the release of energy. On the other hand, an excited singlet state PS can also convert into a more stable, excited triplet state that similarly can revert to the ground state via the aforementioned pathways (Plaetzer et al. 2003). Here, the excited triplet state PS can induce the direct oxidation of cellular molecules and generation of oxygen-containing substrates (Type I reaction), or transfer its energy to molecular oxygen, leading to the formation of highly reactive singlet oxygen (^1^O_2_, Type II reaction) (Przygoda et al. 2023; Maharjan et al. 2022). As a result, these two reactions can react with biological molecules, inducing necrosis from Type I reaction or apoptosis from Type II reaction, which results in the elimination of cancer cells.

However, there are inherent limitations that must be addressed in the current therapeutic approaches to PDT. First, the penetration of light depends on the optical properties of the skin tissue and wavelength used. Tissues and skin surfaces exhibit heterogeneity, causing light to scatter, reflect, transmit, or be absorbed (Gunaydin et al. 2021). In addition, water inside tissue absorbs light at longer wavelengths, limiting the depth of light penetration into the tissue. Thus, it can be expected that long-wavelength light cannot be employed for the excitation of PS because of insufficient light energy for PS to transition into its triplet state and generate singlet oxygen, which results in a decrease in the effectiveness of PDT. On the other hand, endogenous dyes like hemoglobin and melanin absorb light at shorter wavelengths, influencing light penetration (Tuchin 2016). For example, previous research has demonstrated that light with a wavelength below 600 nm can only penetrate 0 to 1 mm from the skin’s surface (Finlayson et al. 2022). Second, the PDT method requires an enhanced targeting ability during the delivery of PS. Conventionally, PS is directly injected into the human body, resulting in its inevitable absorption by healthy cells. As a result, with light application, the radical oxygen produced by PS damages normal cells and tissues. Thus, it is imperative to explore novel approaches and methods to achieve the effective therapeutic treatment of PDT.

Recently, microneedle array patches (MAPs) have attracted considerable attention as alternatives to conventional syringes. Among the several types of MAPs that have been researched, solid-type and transparent MAPs have the potential to enhance light penetration into skin tissue, making them a promising tool for light therapy. Previous literature demonstrated the integration of microneedles and lens array, also known as optical microneedle–lens array (OMLA), to realize the concentration of widely incident light (Kono et al. 2019), and further application of OMLA for localized light delivery to treat melanoma skin cancer (Wu et al. 2022). In addition, we successfully demonstrated selective photothermolysis using OMLA in our previous study (Park et al. 2024).

**Fig. 1:**
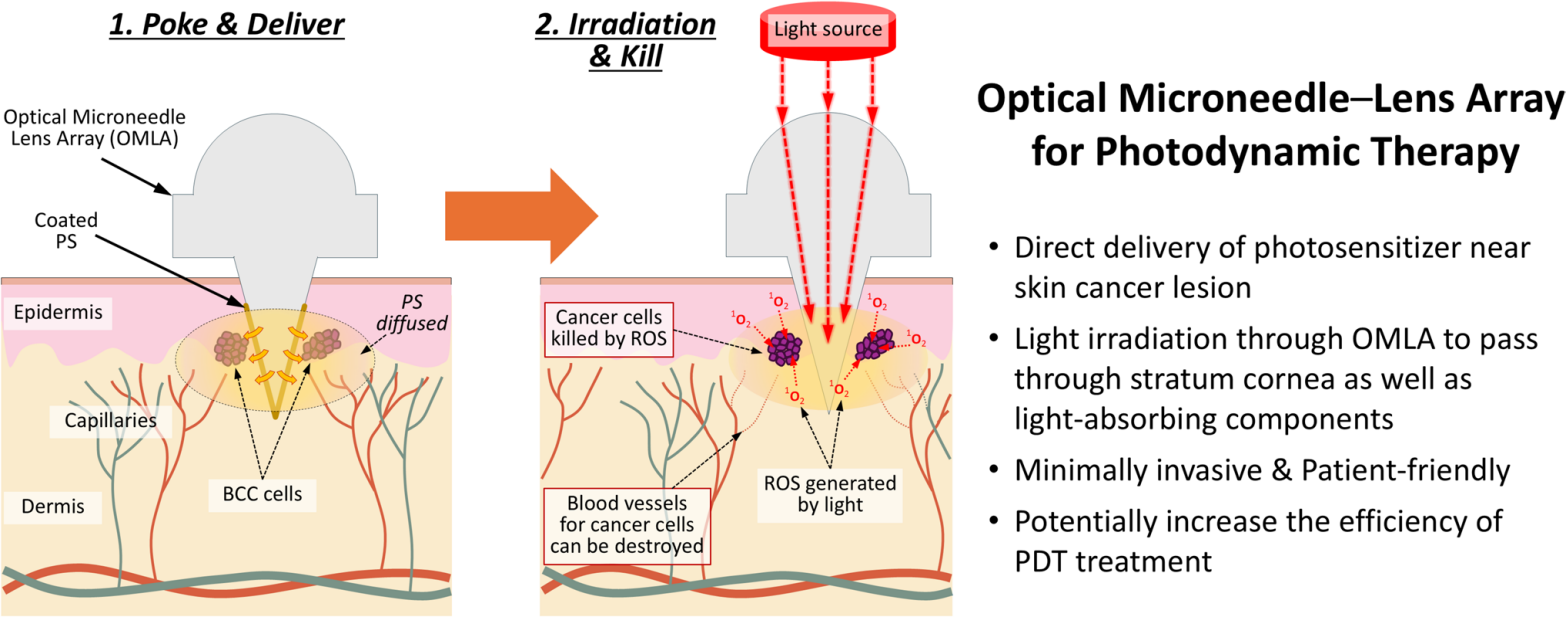
Schematic diagram of the proposed PDT using OMLA in this study

In this study, we propose a novel PDT method using an OMLA with a lens array integrated with microneedles (MNs) in a unit patch. To achieve the PDT effect using an OMLA, we designed an OMLA as a tool for light guidance and direct transmission to a target area inside the skin. In addition, we used an OMLA to deliver PS directly to the target location by coating the surface of the OMLA with PS (Fig. 1). To realize the goals described above, we first performed a computational simulation and optimized the OMLA design to enhance light transmission and achieve effective delivery of PS into a targeted lesion. Next, the OMLA was fabricated using the pin-guided hot embossing method, and the tips of the OMLA were coated with a model drug or PS using the dip coating method. Third, we evaluated the skin penetration and light transmission of OMLA using porcine skin ex vivo. Finally, we confirmed the generation of oxygen species inside an artificial skin model colorimetrically by applying light to the spot where the PS was delivered. From our proposed PDT with OMLA, we expect that PS coated at the tips can be delivered directly to the target area under the skin tissue by the administration of OMLA. In addition, sequential light irradiation onto the lens array of the OMLA activates the photodynamic effect, which results in the release of radical oxygen, thereby eliminating target cancer cells.

## Materials and methods

### Fabrication of optical microneedle–lens array (OMLA)

We used the hot embossing method to fabricate the OMLA based on our previous study (Park et al. 2024). Briefly, we designed and prepared metal master molds for the MNs and the lens array. We then fabricated two different negative molds from each metal master mold using micromolding. Next, we assembled two negative molds using a new alignment method before the hot embossing process instead of the metal cylinder frame used in our previous study. After assembling two negative molds, the OMLA was fabricated using the hot embossing method and polylactic acid (PLA) pellets as a material for OMLA.

Regarding the master mold for the MN array, we designed an MN array with 25 MNs in a square layout of 5 × 5 on a stainless-steel block that was cut into 20 × 20 × 8 mm^3^. The MNs were designed as cone structures with MN base lengths and diameters of 900 and 300 μm, respectively. The center-to-center distances in the horizontal and vertical directions were set as 1.5 mm. In addition, two holes with a diameter of 3 mm for inserting guide pins were drilled in two corners diagonally at 3 mm apart from each edge side.

**Fig. 2:**
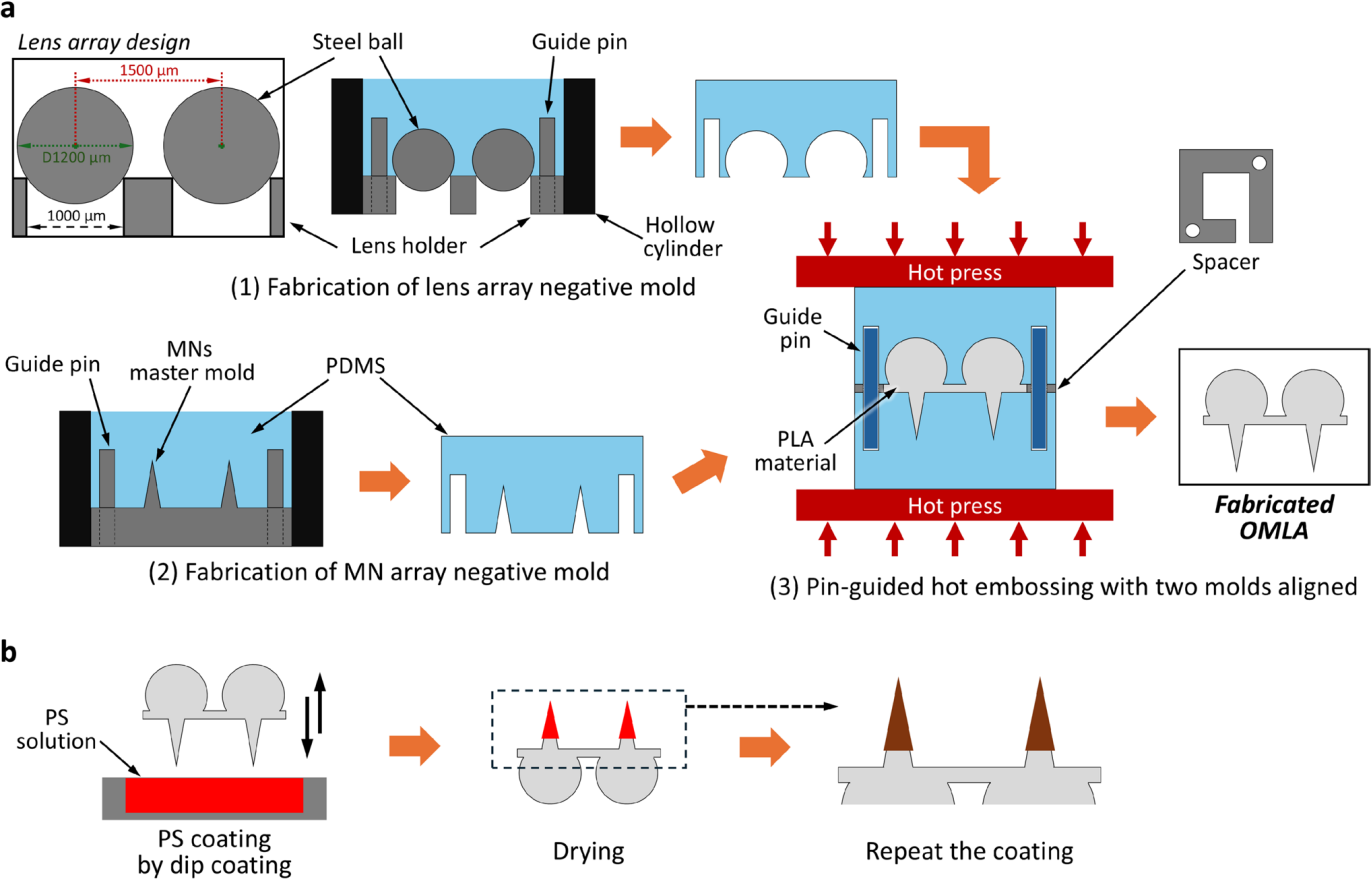
Fabrication process of OMLA used in this study, **a** OMLA fabrication using pin-guided alignment method, **b** dip coating process to coat the surface of MN tips in OMLA

Subsequently, we designed and prepared a microlens holder with holes designated for positioning the steel balls inside. The holder has a square shape with exterior dimensions of 20 × 20 × 2 mm^3^ and 5 × 5 arrayed holes with a diameter of 1 mm in the center. The center-to-center distance for the arrayed holes was set to 1.5 mm as same as the MN array. Steel balls were used to create the space for the spherical lens shape in a negative mold. Here, we used steel balls with a diameter of 1.2 mm (SBM-SUJ-1.2, Tsubaki Nakashima Co., Ltd., Osaka, JAPAN) and positioned them in each hole, resulting in positioning in the same layout as the MN array. Finally, holes for the guide pins were drilled onto the lens array holder with the same diameter and position as the MN mold (Fig. 2).

In addition, we designed a spacer with a dimension of 20 × 20 × 0.8 mm^3^ to keep the final thickness of the OMLA by positioning it between two negative molds during hot embossing. The spacer has an opening with a dimension of 10 × 10 mm^2^ in the center, where the MN array is positioned, and an opening exit with a width of 3 mm for the redundant polymer material (Fig. 2(a3), spacer). All master molds and spacers were fabricated using CNC machining center equipment (Vertical Center Smart 530 C, Yamazaki Mazak Corporation, Niwa, Aichi, Japan) and SUS304 plates (Misumi Group Inc., Tokyo, Japan).

Next, we fabricated negative molds of MNs and a lens array using micromolding. To make negative molds with alignment holes, we prepared two different stainless steel pins with a same diameter of 3 mm and different lengths of 6 mm and 10 mm, respectively (MSHSM3-6 and MSHSM3-10, tolerance: h7, Misumi Group Inc., Tokyo, Japan). Two types of guide pins were inserted into the drilled holes of each master mold before micromolding: 10 mm long pins for the MN array mold and 6 mm long pins for the lens array mold. Here, pins were fixed such that the top 6 mm of the pin was visible for 10 mm long pins and 4 millimeters for 6 mm long pins, respectively. A square-shaped hollow cylinder that fits the metal molds was used to create cubic-shaped negative molds. We positioned the metal molds inside the hollow cylinders and sealed the bottom side using Kapton^®^ tape to prevent leakage during the micromolding process. A mixture of polydimethylsiloxane (PDMS) prepolymer and curing agent (10:1 (w/w), DOWSIL™ SILPOT 184 W/C, Dow Chemical Company, Midland, MI, USA) was then filled inside each cylinder, defoamed at 20 kPa for 1 h, and cured in an oven at 85 ℃ for 1 h. The cured negative molds were released from the master molds and subsequently used for fabrication of the OMLA (Fig. 2(a1, a2)).

We used polylactic acid pellets (3D850, NatureWorks LLC, Plymouth, MN, USA) as the material for OMLA and a heat press machine (H300-01, AS ONE Co., Osaka, Japan) for the hot embossing process. Prior to the hot embossing process, 10 mm long guide pins were inserted into the guide holes of the MN array negative mold in advance. In addition, a spacer was placed on the MN array mold. After positioning the MN array mold with pins and the spacer on the bottom plate of the press machine, 125 mg of PLA pellets were placed inside the opening of the spacer, and the lens array mold was half-assembled while maintaining the distance between the two molds.

Next, the top and bottom plates were heated up to 230 ℃ to melt the PLA pellets and cooled down to 200 ℃ after the pellets had melted completely. The mold set was then fully assembled and pressed at 2 MPa and cooled by turning off the heaters of both the top and bottom plates. The pressure was released after 15 min of initial pressing, and the mold set was left until it cooled down to room temperature. Finally, the mold set was removed from the heat press and disassembled to retrieve OMLA. All the fabricated OMLA were stored in a desiccator until use.

### *Ex vivo* evaluations of fabricated OMLA

We evaluated the penetration ability of the fabricated OMLA using porcine ear skin (K1270, Funakoshi Co. Ltd., Tokyo, JAPAN) as an alternative model to human skin. The porcine skin was taken out from a refrigerator for thawing and kept at room temperature for 1 h before starting the evaluation. The porcine skin was first fixed onto a flat plate using pins at each corner. OMLA was then inserted into porcine skin for 30 s under thumb pressure and removed from the skin. Next, the surface was stained with 1% (w/v) solution of methylene blue (M9140, Merck KGaA, Darmstadt, Germany) for 15 min, and the solution was completely wiped. The penetrated holes were recorded using a digital camera and examined using a digital microscope (VH-5500, Keyence Co., Osaka, Japan). Penetration efficiencies were calculated by dividing the total number of stained spots by the total number of penetrated MNs.

Next, we evaluated the light transmission of the fabricated OMLA by using porcine skin. We used a laser module with a wavelength of 1064 nm (MIL-N-1064, CNI Optoelectronics Tech. Co., Ltd., Changchun, China) and an integrating sphere photodiode power sensor (S142C, range: 350–1100 nm, 1 µW–5 W, Thorlabs, Inc., New Jersey, USA) with a power meter console (PM100D, Thorlabs, Inc., New Jersey, USA). OMLA was inserted into the porcine skin, which was attached to the opening of the power sensor and positioned 10 cm away from the laser source. We measured the light power transmitted through three different samples: porcine skin only, OMLA from our previous study (Park et al. 2024), and OMLA in this study. All transmitted light powers were recorded and plotted for analysis.

### Coating of PS onto the tips of fabricated OMLA

We used a dip coating method to coat the tip of the OMLA with PS solution. To prepare the PS solution for the dip coating method, we first prepared a base solution of 10% (w/v) carboxymethyl cellulose (CMC, 419273, Mw: 90000, Merck KGaA, Darmstadt, Germany) in deionized (DI) water by heating at 90 ℃ with stirring for 1 h. CMC base solution was cooled to room temperature and sealed. 4,4’,4’’,4’’’-(Porphyrin-5,10,15,20-tetrayl)tetrakis(1-methylpyridin-1-ium) 4-methylbenzenesulfonate (TMPyP, A5014, Tokyo Chemical Industry Co., Ltd., Tokyo, Japan) was dissolved in a base solution to a concentration of 10 mM. In addition, we added red dye (025081, Kyoritsu Foods Co., Ltd., Tokyo, Japan) at a concentration of 1% (w/w) to visualize the solution during observation.

Prior to dip coating, the MN surface of the OMLA was treated with air plasma (power: 47 W, time: 160 s) using a benchtop plasma system (YHS-R, SAKIGAKE-Semiconductor Co., Ltd., Kyoto, Japan) to make the surface hydrophilic. OMLA was fixed on an L-shaped holder with MN array facing down at the motorized stage (SGAM(MS)26–100(Z), Sigma Koki Co., Ltd., Saitama, Japan). The prepared PS solution was poured into a plastic dish and positioned below the OMLA. A digital microscope (Dino-Lite, AD7013MTL, AnMo Electronics Co., Hsinchu, Taiwan) was installed to monitor and record the entire dip coating process.

To coat the tips of the OMLA, it was moved 600 μm downward using a stage controller (Mark-204AM-MS, Sigma Koki Co., Ltd., Saitama, Japan) after it touched the top of the PS solution. OMLA was then left undisturbed for 15 s and returned to its original position. Finally, the PS-coated OMLA was fully dried in ambient air for 30 min. The dipping process described above was repeated for the multiple coatings. The fabricated OMLA as well as coated OMLA were observed using a digital microscope (VH-5500, Keyence Co., Osaka, JAPAN / Stemi 305 with Axiocam 105 color, Carl Zeiss AG, Oberkochen, Germany).

### Optical evaluation of PS delivery and the photodynamic effect using artificial skins

To evaluate the PS delivery of TMPyP-coated OMLA, we first used optical microscopy with an inverted optical microscope (IX71, Evident Co., Tokyo, Japan) to visually confirm the delivery of TMPyP into the artificial skin. Regarding the artificial skin, we first prepared an agarose solution by adding an agarose powder (FastGene Agarose, NE-AG01, NIPPON Genetics Co., Ltd., Tokyo, Japan) in DI water at a concentration of 1% (w/v). The mixture was heated inside a microwave to make fully melted agarose solution, poured into a plastic dish. After the gel solution was solidified, the agarose gel was used as the artificial skin. The OMLA coated with TMPyP and color dye was inserted into the prepared gel and removed. The punctured artificial skin was cut, and optical images of cross-sections were taken.

We then used 3,3’,5,5’-tetramethylbenzidine (TMB, 860336, Merck KGaA, Darmstadt, Germany) as a molecular probe to confirm the photodynamic effect that generates reactive oxygen species (ROS) by light irradiation. First, we evaluated the feasibility of TMB as a molecular probe by observing the color change of filter paper functionalized with TMB and TMPyP from OMLA, followed by light irradiation. We dissolved TMPyP that was already coated on the tips of the OMLA by dipping it in 1 mL of DI water for 10 min. Next, we prepared a TMB solution with a concentration of 15 mM in methanol and dropped 10 µL of the solution onto two filter-paper substrates with a diameter of 10 mm, respectively. Subsequently, the same volume of the TMPyP-dissolved solution was dropped onto one of the two substrates. UV light was then irradiated onto all paper substrates for 20 min using a UV LED hand light (M-UVL04, power: 0.5 W, wavelength: 395 nm, MonotaRO Co., Ltd., Osaka, Japan). Color changes of the paper substrates were observed and recorded.

Next, we evaluated the photodynamic effect using PS-coated OMLA and artificial skin. Artificial skin was prepared by mixing 15 mM TMB solution in methanol with a 10% (w/v) gelatin aqueous solution. Here, gelatin aqueous solution was prepared by dissolving gelatin powder (Gelatin Type A, from porcine skin, Nitta Gelatin Inc., Osaka, Japan) in DI water at 80 ℃ with stirring at 300 rpm. Subsequently, the solution was mixed with the prepared TMB solution during cooling down and finally solidified at 4 ℃ in a refrigerator. Once the TMPyP-coated OMLA was inserted into the gelatin, UV light was irradiated onto the OMLA for 20 min. After the OMLA was removed from the skin, puncture holes were observed using an inverted optical microscope (IX71, Evident Co., Tokyo, Japan).

## Results and discussion

### Fabrication of OMLA using pin-guided hot embossing

**Fig. 3:**
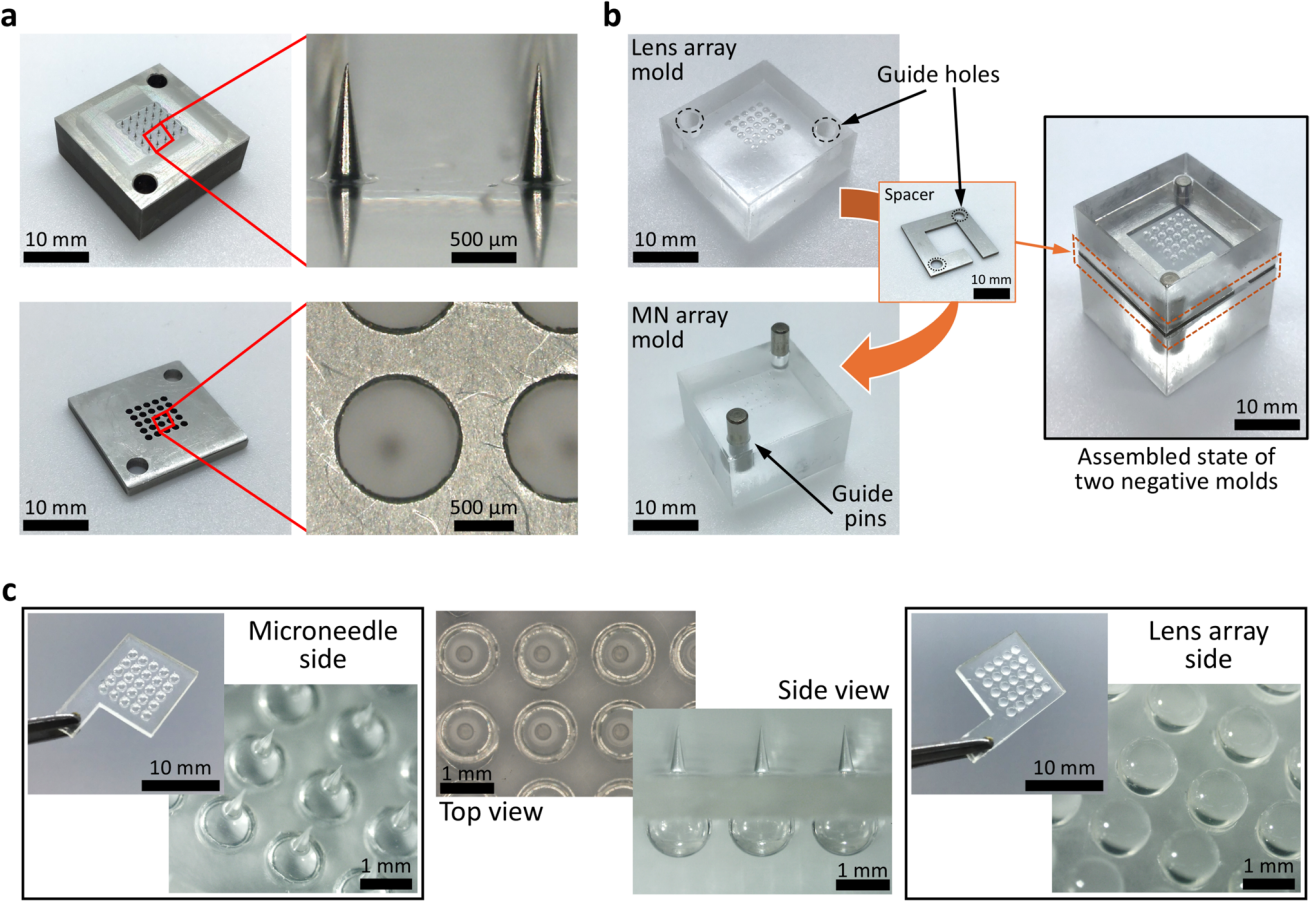
Whole fabrication results of developed OMLA in this study, **a** Fabricated metal master molds for MN array (top) and lens array (bottom), respectively **b** PDMS negative molds fabricated from the master molds and assembled state using a spacer as well as guide pins, **c** Overview of OMLA fabricated by using PLA as a material and hot embossing method, from left to right: optical images from microneedle side, top and side views, and images from lens array side

The fabricated metal master molds for the MN array and lens array are shown in Fig. 3a. The length, diameter, and center-to-center distance of the MNs were 897 ± 9, 297 ± 5, and 1493 ± 7 μm, respectively (*N* = 18 for length and diameter, *N* = 14 for center-to-center distance). In addition, two holes with a diameter of 3.01 mm were drilled in both molds to position the aligning pins. Regarding the lens array holder, the diameter of holes for steel balls and the center-to-center distance were 1034 ± 8 and 1499 ± 3 μm, respectively (*N* = 10 for both dimensions). The spacer that was used for keeping the thickness of OMLA constant was also prepared with the final dimension of 19.99 × 19.99 × 0.8 mm^3^ (Fig. 3b, orange rectangular inset). Here, we determined the optimal thickness of the spacer as 0.8 mm using computational simulation and prepared it as designed (Supplementary Information).

Next, negative PDMS molds were fabricated using metal molds separately. Before PDMS casting, the metal pins were inserted into the respective metal molds to create alignment holes in the PDMS negative molds. As we used 10 mm long guide pins for the hot embossing process, we set the exposed length of pins when they were inserted to 6 and 4 mm for the MN array mold and the lens array mold, respectively. Considering the thickness of the spacer, whose thickness is 0.8 mm, the total expected depth of alignment holes was expected to be 10.8 mm.

Figure 3b shows the fabricated PDMS negative molds, a spacer, and the assembly of both molds. The negative molds of the MN and lens array had dimensions of 20.01 × 20.01 × 12 mm^3^ and 19.99 × 19.99 × 8.45 mm^3^, respectively. The depths of holes by guide pins were 6.04 and 4.42 mm for MN and lens array negative molds, respectively. After inserting 10 mm-long guide pins into the holes of the MN array mold, we positioned the spacer and assembled the lens array mold with alignment by two pins (Fig. 3b). We confirmed that 10 mm long guide pins were fit inside the holes as the depth of the guide holes was calculated 11.26 mm in total by measurement.

OMLA fabricated using the hot embossing method is shown in Fig. 3c. The exterior dimension of OMLA was 10.01 × 10.01 × 0.8 mm^3^ (excluding the handle). We confirmed that the MN parts and lens arrays were successfully fabricated using pin-guided hot embossing. The length, base, and center-to-center distance of the MN array were 880 ± 7, 300 ± 8, and 1493 ± 4 μm (*N* = 18 for length and diameter, *N* = 16 for center-to-center distance), respectively (Fig. 3c, Side view). The thickness of the middle layer of the fabricated OMLA was 801 ± 2 μm (*N* = 8). We confirmed that the dimensions of the fabricated MNs were generally close to those of the metal molds. In addition, we considered that the slight decrease in the length of the MNs as well as the increase in the base were caused by the pressure applied during the hot embossing process. The thickness of the middle layer was close to the optimal thickness based on the results of the computational simulations, as well as the dimensions of the spacer. The diameter of the lens was 1190 ± 10 μm (*N* = 15). Compared to steel balls, we expected that the decrease in diameter was also caused by the pressure in the same manner as the MN arrays. Thus, we considered that the dimensional difference between the design and the fabricated OMLA can be minimized by finding optimal pressure during the hot embossing process.

Finally, the misalignment of the MNs and the lens was measured using an optical microscope (Fig. 3c, Top view). The distance between the centers of the MNs and lenses was 41 ± 10 μm (*N* = 12). Compared with our previous study using frame-guided hot embossing, the misalignment decreased from 52 to 41 μm. As a result, we considered that the newly proposed pin-guided hot embossing method was effective in decreasing misalignment when using the assembly of two different negative molds. Simultaneously, we confirmed that the thickness of the middle layer was successfully achieved by introducing a spacer. As the light propagates through all OMLA structures with being influenced by their dimensions, it is considered important to fabricate OMLA with more precise dimensions. For example, increasing the number of guide pins can be a feasible measure to reduce the alignment error between the MNs and lens array. In addition, the optimized hot embossing process with precisely controlled pressure, temperature, and time is expected to significantly reduce the dimensional error and increase the reproducibility.

### *Ex vivo* evaluations of fabricated OMLA regarding penetration ability and light transmission

**Fig. 4:**
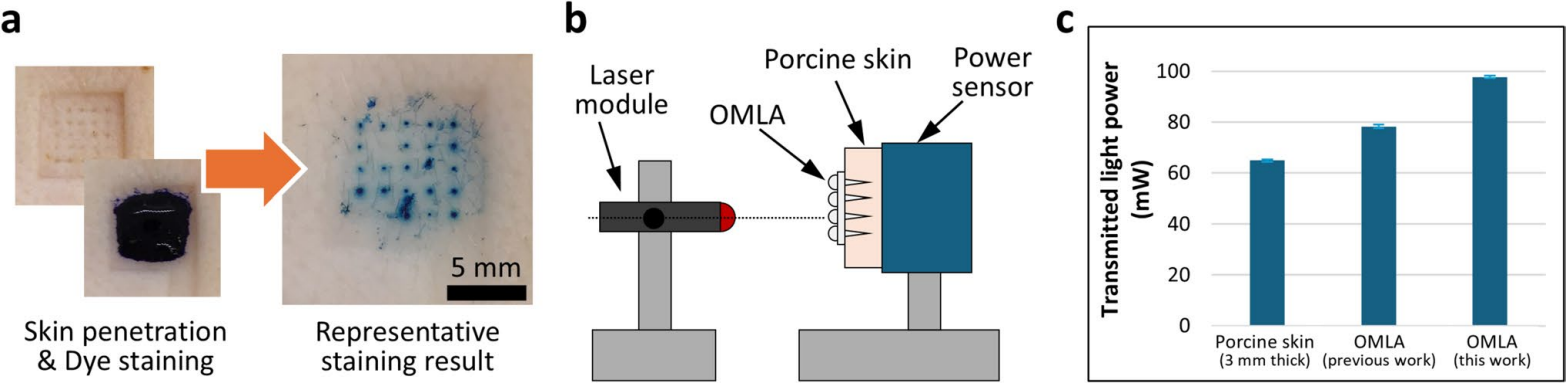
*Ex vivo* evaluations of fabricated OMLA, **a** Skin penetration evaluation using a porcine skin **b** Schematic diagram of the light transmission evaluation setup, **c** The power of transmitted light with respect to different experimental conditions: porcine skin only, OMLA at previous study (Park et al. 2024), and OMLA fabricated in this study (N=5 for each case)

We investigated the penetration ability of the fabricated OMLA using porcine skin, expecting its practical use in clinical treatment. As previous studies have already reported that PLA-based MNs have sufficient mechanical strength to penetrate skin tissues (Wu et al. 2022; Kang et al. 2022), we performed an *ex vivo* evaluation only in this study using the fabricated OMLA. In addition, porcine skin was used for *ex vivo* evaluations to mimic human skin because of its anatomical and physiological similarities (Summerfield et al. 2015). From the penetration evaluation, we achieved a successful skin penetration rate of 90.4% on average (*N* = 5, Fig. 4a). From these results, we consider that the fabricated OMLA is sufficiently rigid to penetrate real skin tissue.

Next, we evaluated the light transmission through the OMLA inserted into the skin. Figure 4b shows the schematic of the experimental setup. The light travels from the light source to the sensing part of the power sensor sequentially through OMLA and skin. In the case of the skin only, which was used as a control, the power of the transmitted light was 64.8 ± 0.4 mW (*N* = 5, Fig. 4c). Next, we used the OMLA proposed in the previous study and obtained a 78.2 ± 0.8 mW of transmitted light power (*N* = 5). The previous OMLA had a middle layer with a thickness of 889 ± 24 μm, which was not precisely controlled during its fabrication. On the contrary, the thickness of the OMLA in this study had an optimal thickness of 801 ± 2 μm, which was calculated from computational simulations and was fabricated by using a spacer with the designed thickness. By using the OMLA developed in this study, we achieved 97.7 ± 0.5 mW (*N* = 5), which results in 25% increase over our previous OMLA. From the results, we confirmed that the transmission of light was improved by introducing the optimized thickness of a middle layer from the computational simulation. In addition, we confirmed that the results agreed well with the simulation results, which included an increase in light transmission with a decrease in the thickness of the middle layer (Fig S5b, Supplementary Information).

### Coating of PS onto the tips of fabricated OMLA

Regarding the delivery of PS using OMLA, we considered coating the surface of the PS material to be one of the most practical and effective methods. Once the tips of MNs are coated with water-soluble drug material and inserted into skin tissue, the drug will dissolve by body fluids present in the skin, and then diffuse into the surrounding tissue without any external forces. Among several surface coating methods, we chose the dip coating method in this study because of its simplicity and processability. In addition, we chose TMPyP as a PS because of its usability for PDT, negligible dark toxicity, excellent water solubility, and rapid clearance from the organism (Eckl et al. 2018). Also, TMPyP is known to have an absorption maximum in the wavelength of around 420 nm (Eckl et al. 2018; Schulz et al. 2022). As a thickening agent as well as a base solution for the PS solution used in the dip coating, we used CMC as it is widely used for various applications in conventional drug delivery owing to its biodegradability.

**Fig. 5:**
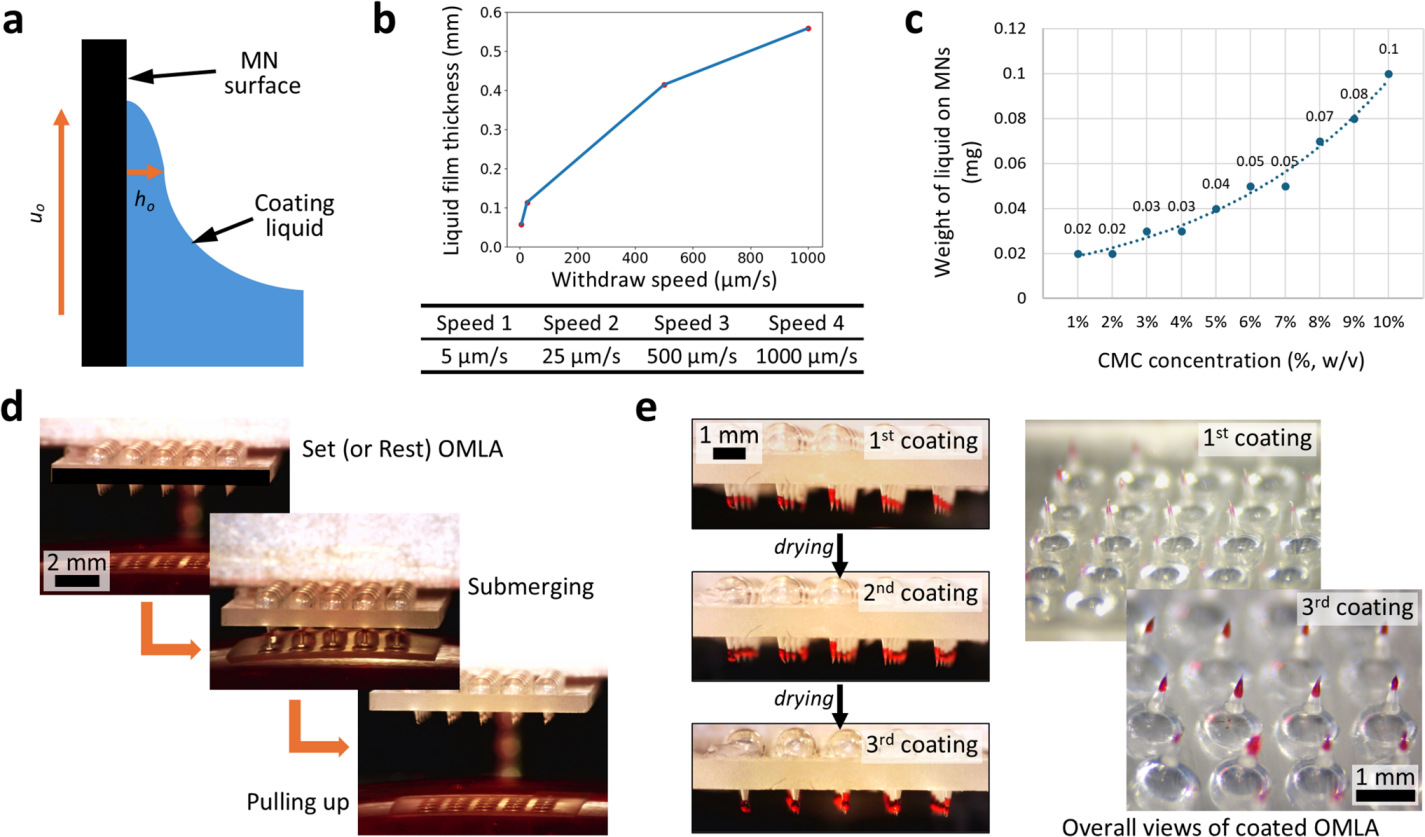
Dip coating of PS onto OMLA and multiple coating results, **a** Schematics of dip coating process, **b** Relationship between the thickness of liquid film and withdrawing speed, **c** Weight measurement of coated solution on OMLA with respect to different CMC concentration, **d** Optical observation of dip coating process including three steps, **e** Microscopic images of multiple coating processes

Before the dip coating process using OMLA, we performed a theoretical review by simplifying the dip coating process. We assumed a dip coating with a vertical plate, speculating that the side wall of the MNs was withdrawn from a coating liquid reservoir (Fig. 5a). Considering the composition of the PS solution, we assumed that the physical properties of the solution are those of a CMC aqueous solution. According to the previous literature (Chaim and John 1965), the thickness of the liquid film deposited on the microneedle, supposed to be a flat plate in this study, can be represented as

$$\:{h}_{o}={\left[{\left(\frac{2n+1}{n}\right)}^{2n}{\left(\frac{\alpha\:}{\sqrt{2}}\right)}^{3}\frac{{K}^{2}{{u}_{o}}^{2n}}{\sqrt{\sigma\:}{\rho\:g}^{3/2}}\right]}^{\frac{1}{2n+1}}$$

, where $\:{h}_{o}$ is the film thickness, $\:n$ is the flow behavior index, $\:\alpha\:$ is the constant, $\:K$ is the flow consistency index, $\:{u}_{o}$ is the withdrawal speed, $\:\sigma\:$ is the surface tension at the liquid-air interface, $\:\rho\:$ is the density, and $\:g$ is the gravitational acceleration. From the equation, it can be inferred that the thickness of the liquid film formed on the microneedle is primarily influenced by the withdrawal speed, $\:{u}_{o}$, and the inherent properties of the coating liquid.

We set four different withdrawal speeds, including 5, 25, 500, and 1000 μm/s, and then calculated the thickness of the liquid films theoretically using the expression above and previously reported values of CMC solution (Edali et al. 2001). From the calculation results shown in Fig. 5b, we confirmed that the thickness of the liquid film increased as the withdrawal speed of the MNs increased, which resulted in the coating of more liquid on the surface of the MNs. For the dip coating process, we finally chose 1000 μm/s as the withdrawing speed based on the calculation results to achieve the maximum coating amount.

Subsequently, we experimentally evaluated the amount of liquid on the MN surface with respect to CMC solutions at different concentrations. Here, we used from 1 to 10% (w/v) CMC solutions and measured the weight difference between before and after each dip coating step. From these results, we confirmed that the weight of the coated liquid increased as the concentration increased. In addition, the exponential relationship was confirmed by fitting all results, as shown in Fig. 5c. To achieve the maximum amount of coated liquid while considering solution handling, we chose a 10% (w/v) CMC concentration for further evaluation.

For the dip coating process, we used 10 mM TMPyP solution with 10% (w/v) CMC solution as a base. In addition, we added red dye for clear visualization during microscopic observations. Regarding the dip coating process, we considered coating all tips on the OMLA for each coating process to obtain as uniform a coating performance as possible. Thus, we used a flat dish with a diameter of 90 mm and spread the solution after adding solution so that the solution surface becomes as flat as possible. In addition, the OMLA surface was treated with air plasma prior to dip coating to improve wetting, as PLA is known to have a hydrophobic surface.

In this study, we performed the dip coating process with three main steps: setting (or resting) the OMLA, submerging the OMLA with a designated depth, and pulling up (Fig. 5d, Supplementary Video). Although a slightly curved surface of the PS solution was observed due to surface tension, we successfully coated the tips of the OMLA using a precisely controlled stage, as described in the Methods section (Fig. 5e, top). Here, we set the downward movement to maximum 600 μm after the tip of the OMLA first touched the surface of the PS solution in the center part, as the longer movement caused the liquid to climb up by surface tension to the surface of the middle layer. From the results, it was observed that the vertical lengths of coated parts were not uniform over the MN array because of curved surface in the PS solution. As a result, it was expected that the uneven length of the coated parts could cause uneven distribution of PS after OMLA application. Thus, further consideration to make the surface of PS solution flat and the subsequent optimization of a solution vessel are necessary to achieve uniform coating of the PS solution as well as reliable dip coating of the PS solution.

Next, we applied multiple coatings of PS onto the OMLA tips to achieve the maximum loading capacity using the dip coating method. In the aspect of drug capacity, a single coating method might fail to meet the demands for drug capacity in practical applications. For multiple coatings, the dip coating process was repeated after each coating was completed. Figure 5e shows the results for each step of the dip coating process. We observed that fresh liquid droplets were coated onto the surface of the previously coated layer, and the color of the PS solution became darker and thicker with repeated coating. Following each coating cycle, the average weight change of OMLA was measured as 0.08 mg per coating cycle. From the concentration and weight changes, we expected that approximately 3.3 µg of TMPyP would be coated onto the tips of the OMLA. To predict the effect of therapeutic treatment precisely, we considered that spectrophotometric analysis is necessary to quantify the amount of coated PS with respect to the number of coating cycles.

### Evaluation of PS delivery and photodynamic effect using artificial skins

**Fig. 6:**
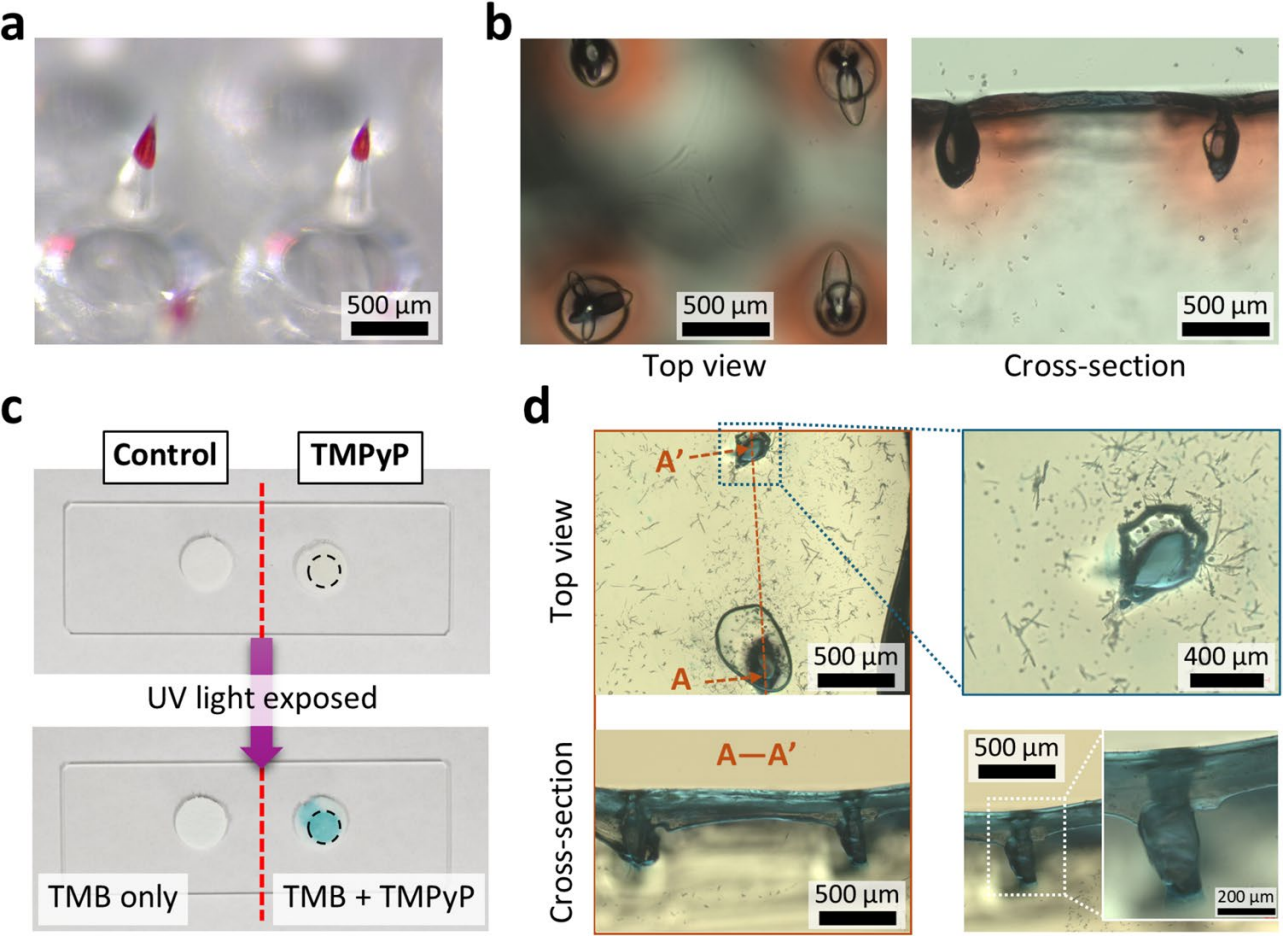
Evaluations of PS delivery and photodynamic effect by OMLA, **a** PS and color dye coated tips of OMLA, **b** Evaluation results of PS delivery into an artificial skin, **c** Feasibility evaluation of TMB as a molecule probe for the photodynamic effect, **d** Optical observation of puncture holes by OMLA after light irradiation

Next, we evaluated the PS delivery of OMLA using an artificial skin. Using OMLA coated with red dye, as shown in Fig. 6a, we observed the diffusion of color dye into the skin using optical microscopy. Figure 6b shows the top view and the cross-section of the punctured parts using the coated OMLA in the artificial skin. The diffusion of the color dye was confirmed by the color change on the surface and inside the skin.

Along with the results of PS delivery into the artificial skin, it was also necessary to determine whether the developed OMLA device could initiate photodynamic effects with light irradiation. Thus, we evaluated the photodynamic effect of PS delivery using a PS-coated OMLA device. To confirm the photodynamic effect, we focused on reactive oxygen species (ROS) in photodynamic therapy which essentially necessitates the generation of ROS from PS as well as light irradiation to achieve specific treatment effects such as the destruction of cancer cells. Previous studies have reported the effectiveness of TMB as a suitable probe for ROS detection (Bresolí-Obach 2020). The working principle is that ROS can oxidize TMB, resulting in the formation of a blue-colored intermediate known as a charge-transfer complex. As a result, we used the color change caused by the reaction of TMB and ROS to detect ROS generation, followed by confirmation of the photodynamic effect of a PS-coated OMLA.

First, we evaluated the feasibility of TMB as a photodynamic probe using a colorimetric method with dissolved TMPyP from the tips of a coated OMLA. Figure 6c shows the results before and after the UV irradiation. We clearly observed the spot where the TMB solution was absorbed underwent a color change from white to blue. Thus, we concluded that TMB can be effectively used to evaluate the photodynamic effects of TMPyP-coated OMLA. Next, we applied a TMPyP-coated OMLA onto artificial gelatin skin and irradiated it with UV light to initiate a photodynamic effect. Figure 6d shows the microscopic images of the artificial skin after removing the OMLA. The top-view images confirmed that the OMLA penetrated the skin successfully. In addition, we observed that the spot where the MNs of OMLA penetrated had a clear blue color, which suggests the generation of ROS from dissolved TMPyP with light irradiation and subsequent color change of TMB on site (Fig. 6d, blue rectangle). Cross-sectional images also confirmed that the entire MN-penetrated spots had color changes, suggesting a photodynamic effect (Fig. 6d, A-A’ section, red rectangle). However, it was observed in the result photos that the surface between two penetrated spots exhibited color changes after cutting in half for the observation of cross-sections. It was expected that dissolved TMPyP as well as TMB was pressed and spread along the skin edge when the cut surface was moved onto a glass slide facing downwards during the observation using an inverted microscope. We also considered that using a blade for cross-sectional images caused the accidental spread of TMPyP and TMB by the blade movement. Thus, we expected that an additional method such as confocal microscopy would be necessary to obtain the cross-sectioning of an artificial skin without changing the sample shape or conditions.

As a result, we successfully verified the photodynamic capability of the TMPyP-coated OMLA. We believe that the experiments and evaluations performed in this study provide a comprehensive validation of the functionality and potential therapeutic applications of the OMLA. At the same time, we consider that further evaluations, such as quantitative analyses regarding the amount of coated PS during the dip coating process and the amount of delivered PS during skin penetration are necessary to predict the efficiency of PDT treatment and optimize the PDT treatment using the proposed OMLA.

Meanwhile, the feasibility of OMLA should be also considered based on the depth of targeted lesions. Longer or shorter MNs are expected to be necessary to deal with various targets at different depths appropriately as the light should be delivered near the target cells, e.g. skin cancer cells, with diffused PS to initiate photodynamic effect. Considering the lengths of MNs that were fabricated in our previous works (Kono et al. 2019; Wu et al. 2022; Park et al. 2024) and the simulation result (Fig S3, Supplementary Information), we consider that the effective depth in using OMLA ranges up to 1.5 ~ 2 mm approximately. Thus, it is expected that the proposed OMLA for PDT can be effectively used to deal with target cells in epidermis as well as dermis layer to achieve therapeutic treatment.

## Conclusion

In this study, we developed and evaluated OMLA for skin penetration, light transmission through skin tissue, PS delivery, and photodynamic effects to propose a new PDT method using OMLA. We designed an OMLA using computational modeling techniques and realized it using a pin-guided hot embossing method. We achieved a higher light transmission efficiency compared to previous study and a skin penetration capability of over 90% using the fabricated OMLA. The dip coating method was performed to coat PS onto the tips of OMLA and a coating of around 3.3 µg of PS, TMPyP, was achieved via multiple coating processes. Finally, we confirmed that our developed OMLA can initiate the photodynamic effect, including the generation of ROS, colorimetrically from the application of TMPyP-coated OMLA onto artificial skin and subsequent light irradiation. We believe that our developed OMLA can be used as a new and efficient tool not only for advancing light-based therapies, but for various skin cancer treatments. At the same time, we expect that further research will be necessary for further enhancement of OMLA, considering quantitative analyses of PS in terms of coating amount, delivery amount, and efficiency, as well as PDT effectiveness compared to conventional PDT. Furthermore, it is expected that the evaluation of OMLA combined with various PSs, such as 5-aminolevulinic acid (5-ALA) and Rose Bengal, can accelerate the introduction of OMLA based PDT in clinical fields.

## Supplementary Information

Below is the link to the electronic supplementary material.

ESM 1 (DOCX 2.60 MB)

ESM 2 (WMV 12.1 MB)

## Data Availability

No datasets were generated or analysed during the current study.
