# Supplementary material for: Development of optical microneedle–lens array for photodynamic therapy
Source: Biomed Microdevices. 2025 Jan 28;27(1):6. doi: 10.1007/s10544-025-00735-4 (PMC11775042; doi:10.1007/s10544-025-00735-4)
Supplement: Supplementary file 1 — (DOCX 2.60 MB) [file 10544_2025_735_MOESM1_ESM.docx]

**Supplementary Information**

**Development of** **optical microneedle–lens array for photodynamic therapy**

Jongho Park^1^, Jingzong Zhang^2^, and Beomjoon Kim^1,*^

1 Institute of Industrial Science, The University of Tokyo, Tokyo 153-8505, Japan

2 Department of Precision Engineering, School of Engineering, The University of Tokyo, Tokyo 113-8656, Japan

*Correspondence: bjoonkim@iis.u-tokyo.ac.jp; Tel.: +81-3-5452-6224

**COMPUTATIONAL SIMULATION FOR OPTICAL MICRONEEDLE–LENS ARRAY**

**1 Simulation about relative energy deposition and fluence rate using Monte Carlo simulation**

**1.1 Introduction**

Regarding PDT, the propagation of light is one of most critical parameters over all considerable factors. At wavelengths between 400 nm and 600 nm, minimal light can penetrate to a depth of 1 mm beneath the skin. Although red light-based photosensitizers are in use, it results in the ineffectiveness of short-wavelength photosensitizers due to their poor performance (Evan et al. 2021). To solve this issue, we proposed the optical microneedle–lens array (OMLA) for PDT in this work to enhance the light transmission. Thus, we first implemented a computational simulation method to expect the light transmission and its characteristics of OMLA to realize OMLA as a new tool for PDT.

Among several models for computational simulations, we first chose Monte Carlo simulation as it allows for the definition of skin tissues consisting of several layers, subsequent calculations, and the accurate reflection of energy distribution. In addition, Monte Carlo simulation method is widely employed in the engineering field and is regarded as the standard for studying light propagation within biological tissues (Vijitha and Manojit 2017).

The Monte Carlo simulation is a method predicated on repeated random sampling and statistical analysis to yield computational calculation results. As a result, it is intimately associated with random experimentation. In Monte Carlo simulations, samples are randomly drawn from a defined statistical distribution to serve as variables for the mathematical model. Outputs corresponding to these inputs can be obtained individually. The subsequent analysis of these output parameters allows for the derivation of conclusions.

Generally, Monte Carlo methods can be broadly divided into two categories. The first category includes problems that inherently exhibit intrinsic randomness. Such problems can be directly simulated using the computational capacities of contemporary computers. The second category encapsulates problems that can be transformed into features of a random distribution. This might include the likelihood of the occurrence of a stochastic event or the expected value of a random variable. These transformed problems can then be effectively addressed through the application of Monte Carlo methods.

Regarding the use of Monte Carlo simulation about the light transmission in biological tissues, it was supposed that substantial quantity of photons is propagated within a simulated medium that is typically composed of several tissue layers. During their propagation, these photons experience a variety of optical phenomena, including reflection, refraction, absorption, and scattering. In this work, we used open-source Monte Carlo simulation codes and MATLAB (The MathWorks, Inc, MA, USA) to expect light transmission and energy in using OMLA via the design of OMLA, the definition of tissue layers as well as OMLA, and the energy distribution during light transmission through OMLA. We used the open-source codes provided by the Oregon Medical Laser Center (OMLC) (Prahl 2024), with the calculation method detailed in 3D Monte Carlo simulation of heterogeneous tissues section. Here, we generally used all predefined values about tissue parameters to expect light propagation and energy deposition using OMLA approximately.

**1.2 Definition of OMLA dimensions**

To proceed with the simulation, we first define the dimensions of OMLA. Given that non-melanoma tumor tissue is predominantly localized within the epidermal and dermal layers of the skin, the application of OMLA is crucial for the targeted delivery of excitation light and photosensitizers to the dermis layer. The dimensions of the microneedles (MNs) and microlenses were determined based on specific requirements and considerations.

Considering the dimensions of OMLA, the total length of the MN should exceed the thickness of the skin layer it needs to penetrate. However, previous research has revealed the presence of air gap, ranging from 100-200 µm that hinders the complete insertion of MNs into the skin (Makvandi et al. 2021). Thus, we defined the dimensions of MNs in OMLA so that the overall length exceeds both this air gap length and the thickness of the various skin layers that were previously referred (Park et al. 2024). In addition, it was reported that the depth of non-melanoma cancer cells in early stage varies from 170 to 400 µm (Pyne et al. 2018). As a result, we could have the following equation to ensure efficient MN penetration:

$$Length of MN>\Sigma( Air gap, Thickness of stratum corneum and epidermis layer )$$

From the consideration above, we set the MN’s length as 900 µm, expecting it can penetrate the stratum corneum layer as well as epidermis layer, and finally reach the papillary dermis where the target exists.

| 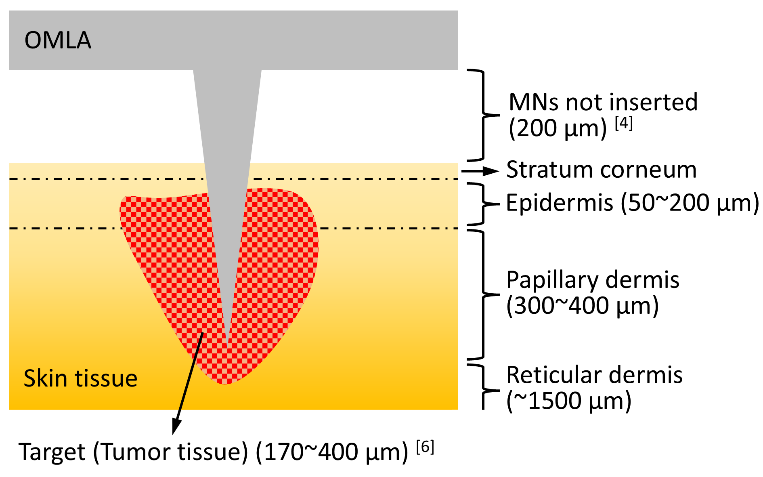  **Fig. S1** Definition of skin layers with OMLA. |
| --- |

The vertex angle of MNs was adjusted so that it is less than 30° to minimize pain as well as discomfort during OMLA insertion and reduce the force required for insertion. The base of MNs was calculated based on the vertex angle and the length of MNs. Regarding the center-to-center distance between each MN in the array, we set it 1.5 mm to facilitate MNs’ insertion. Based on the conditions and consideration described above, we finally set the dimensions of OMLA to ensure that the incident light of the OMLA converges near the tips of the microneedles as shown in Table S1.

**Table S1** Determined dimensions of MNs and microlens.

| **Parameters** | **Dimensions** |
| --- | --- |
| Microneedle pitch | 1500 µm |
| Length of microneedle | 900 µm |
| Diameter of microlens | 1200 µm |
| Tip angle | 30° |
| Resultant tip area | 10 µm^2^ |

**1.3 Definition of skin tissue layers and MN**

Basically, we used all pre-defined optical parameters and tissue structure values from the provided code package, including scattering and absorption coefficients, in skin tissue designs. In addition, it was necessary to determine additional optical parameters that are specific for OMLA in this work. Thus, we added the information related to polylactic acid (PLA) material into the code (corresponding code file: makeTissueList.m) as the MN material in work is PLA. Referring to the previous literature about PLA material (Mikołajczyk and Kuberski 2019), we set the necessary parameters and included to the simulation codes as follows: layer name = PLA microneedle, absorption coefficient = 0.001, scattering coefficient = 13, anisotropy of scattering = 1. Here, all values were set based on a wavelength of 400 nm from the previous literature. After defining the default skin tissue layer, we also defined the skin model with an inserted MN using the determined dimensions. Fig. S2 shows the finished skin definitions without MN (left) and the skin definition with MN penetrated (right), respectively, which were used through Monte Carlo simulation in this work.

| 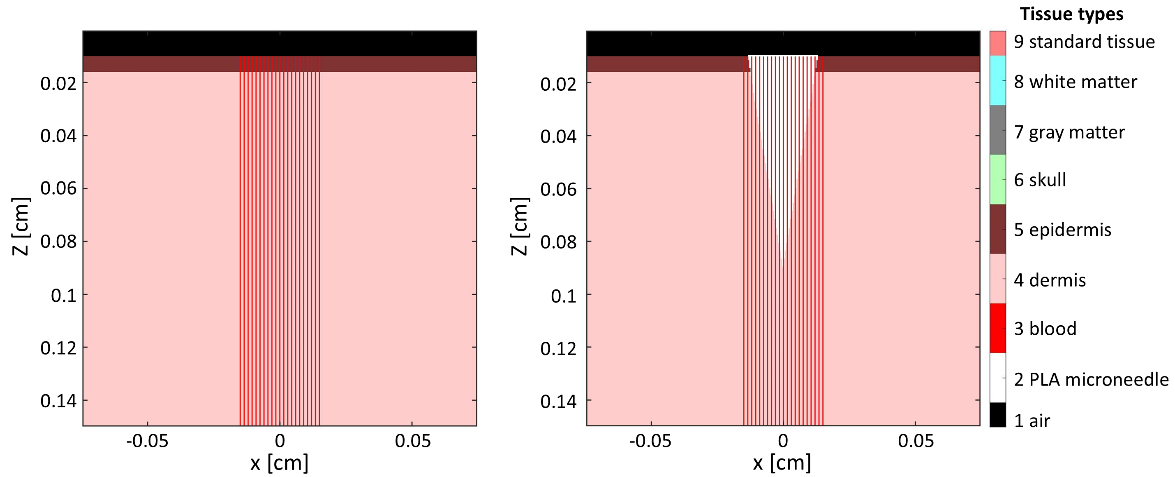  **Fig. S2** Definition of skin tissue designs without (left) or with MN (right). |
| --- |

**1.4 Simulation for skin energy deposition**

Next, we ran the simulation using determined parameters above for a duration of 10 minutes, which resulted in the computation of 825,219 photons in total. Here, we simulated the relative energy deposition and the relative fluence in the skin layers for both cases, without or with MN. In addition, incident light was designed as circular blue spots, with a wavelength of 400 nm, and was irradiated on the skin surface.

Fig. S3 shows the simulation results about the relative energy deposition as well as the relative fluence rate. From the results, we could confirm that the implementation of OMLA, *i.e.* using a MN structure, considerably amplified the light energy within the skin compared to that without MN. Importantly, we observed around tenfold enhancement in light energy deposition with MN compared to the skin without MN within a depth range of 1.5 mm. Although we used the predefined parameters to expect approximate results with MNs, we expect that current results sufficiently showed the improved efficiency of energy deposition as well as relative fluence with MN structures. In conclusion, we confirm from Monte Carlo simulation results that the implementation of OMLA, with MN structures, can enhance the transmission of light energy into the deeper region compared to that without OMLA, *i.e.* conventional light therapy. Thus, we expect that our developed OMLA with a MN array can improve the efficiency of PDT even with the tumors that exist in deeper regions.

| 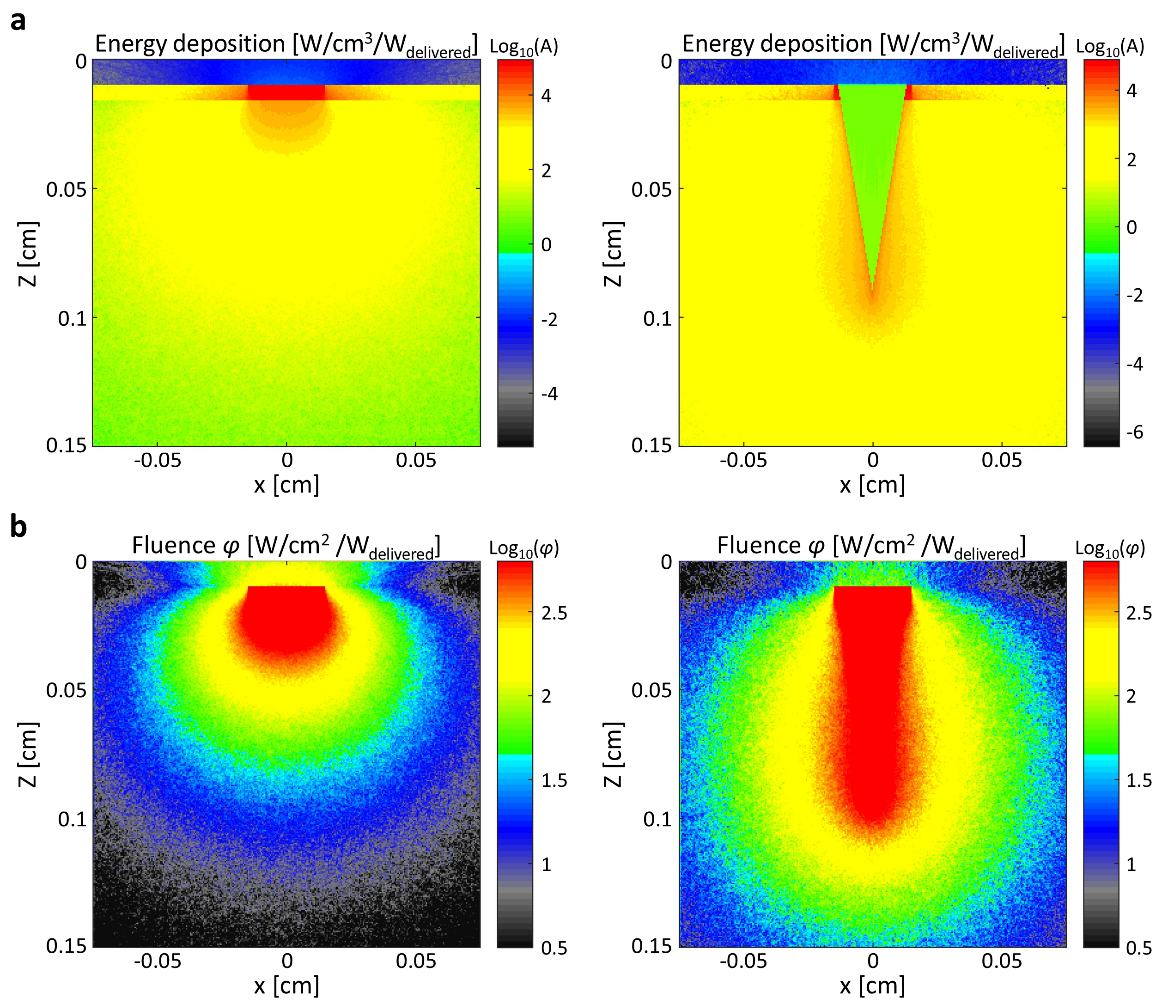  **Fig. S3** Monte Carlo simulation results, (a) Mapping results of relative energy deposition without MN (left) and with MN (right), (b) Results about relative fluence without MN (left) and with MN (right). |
| --- |

**2 Optimization of the middle layer thickness in OMLA using COMSOL**

**2.1 Introduction**

For the design of OMLA in our previous work (Park et al. 2024), we performed computational simulations to analyze the trajectory of light, changes of light transmission with different layer thicknesses and different incident angles. However, we changed the dimension and the layout of MNs or microlenses considering PDT application in this work. Among the dimensional changes, we focused on the thickness of the middle layer in OMLA as it was not inaccurately controlled in our previous work. The middle layer of OMLA, the layer situated between MN and microlens, has an important role in transmitting light from lens side to MN side. As a result, the inaccurate control of its thickness can lead to unexpected results being different from theoretical calculations regarding light transmission. Thus, we intended to optimize the thickness of the middle layer in OMLA along with the changed dimensions in this work.

Similar to the previous work, we used the ray tracing module in COMSOL software (version 5.6) to analyze the trajectory of each photon in light propagation. We set simulation parameters of skin tissue, OMLA including MNs and lens, and relevant configuration for calculations in order. Here, we mainly describe the changes or different points compared to the details of previous simulations.

**2.2 COMSOL Simulation configuration**

First, we defined the optical properties involved in the simulation. Regarding the wavelength, we used the same value, 400 nm, as that used for Monte Carlo simulation. In addition, the refractive index of PLA, *i.e.* OMLA material, was defined as 1.52 at a wavelength of 400 nm (Bodurov et al. 2016). The material for all PLA parts was set as the predefined PLA material, (C3H4O2)n (Polylactic acid, PLA) (Bodurov et al. 2016: PDLA; n 0.405-0.635 μm), in the database of COMSOL application. Also, the refractive index of the skin layer was set as 1.41 representatively (Lister et al. 2012). The refractive index of the peripheral space and ambient air were assigned a value of 1. Regarding the dimensions for OMLA, we used the values shown in Table S2. Subsequently, the dimensions of the model were defined, as presented in Fig. S4a, with millimeters being used as the unit of measurement.

**Table S2** Parameters used in the simulation model.

| **Name** | **Expression** | **Value** | **Description** |
| --- | --- | --- | --- |
| h1 | 0.8 [mm] | 8E−4 m | Thickness of OMLA |
| r1 | 0.6 [mm] | 6E−4 m | Radius of a microlens |
| r2 | 0.5 [mm] | 5E−4 m | Radius of jig |
| dis | 0.0 [mm] | 0 m | Misalignment |

Regarding the light source, we set the light source to 'release from grid', with a grid consisting of 100 uniformly distributed x-axis coordinates. The direction of transmitted light was set vertically downwards by setting a y-directional vector value to -1. Consequently, 100 beams of light were incident from above the microlens, distributed uniformly across the horizontal plane as shown Fig. S4b.

Next, we defined the parametric sweep parameters to carry out individual computational simulations for different parameters. This sweep resulted in a set of computational results influenced by varying parameter values, simplifying comparative analyses and allowing us to discern the impact of each parameter on the entire system. Here, we set the parametric values for the thickness of the middle layer ranging from 0.4 to 1.2 with increasing 0.1.

In addition, we set the termination condition for the simulation by setting the lengths of ray tracing to 10 mm, which is sufficient to encompass the entire model. Finally, we designed the photon counter, assuming the tumor tissue, so that it quantifies the number of residual rays within our model to get the representative value for the number of light rays reaching the target tissue (Fig. S4b, photon counter).

| 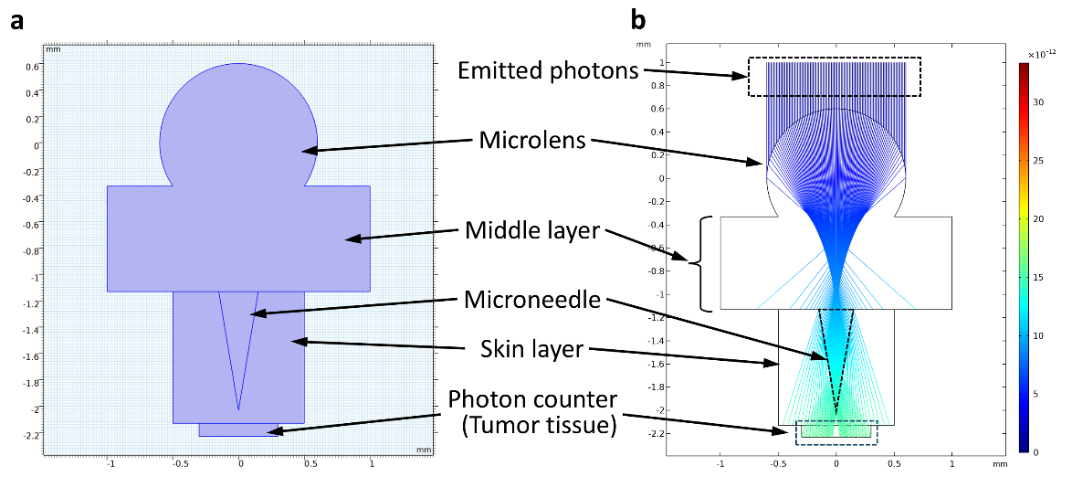  **Fig. S4** Simulation setup and results, (a) Geometry of OMLA setup in COMSOL, (b) Representative ray trajectory simulation results with a 0.8 mm-thick middle layer. |
| --- |

**2.3 Ray tracing simulation and analysis**

Fig. S5a shows the ray tracing simulation results with different thicknesses of a middle layer. From the result, it was confirmed that 100 parallel photons are emitted towards the OMLA from the upside. A photon counter is situated at the bottom to measure the number of photons that reach the assuming tumor area during the PDT process. Here, the thickness of the middle layer between the microlens and the microneedle equates to the thickness of the OMLA, which can be a parameter that evidently impacts OMLA's optical performance.

To determine the optimal OMLA thickness, we first evaluated the light transmission efficiency of the OMLA. From the COMSOL results, we calculated the efficiency using the following equation and the photon numbers collected at photon counter:

$$Light transmission efficiency=\frac{Number of photons received}{All emitted photons}$$

Fig. S5b shows the relationship between light transmission efficiency and the thickness of the middle layer. We observed that light transmission efficiency decreased as thickness became thicker. It was considered that the light dissipated to surroundings as the locational change of the focal point which was caused by increased thickness. In the chart, we had a plateau in light transmission efficiency when the thickness is in the range from 700 to 800 µm. Although we could get more increase in light transmission efficiency with decreasing thickness, the increase rate was relatively marginal when compared to a thickness of 800 µm. Considering the error in the fabrication process, the hot embossing method, and the increase rate with decreasing thickness, we concluded that the range of 700–800 µm is appropriate and practical. Finally, we set the thickness of the middle layer in OMLA to 800 µm and designed the spacer with the determined thickness.

| 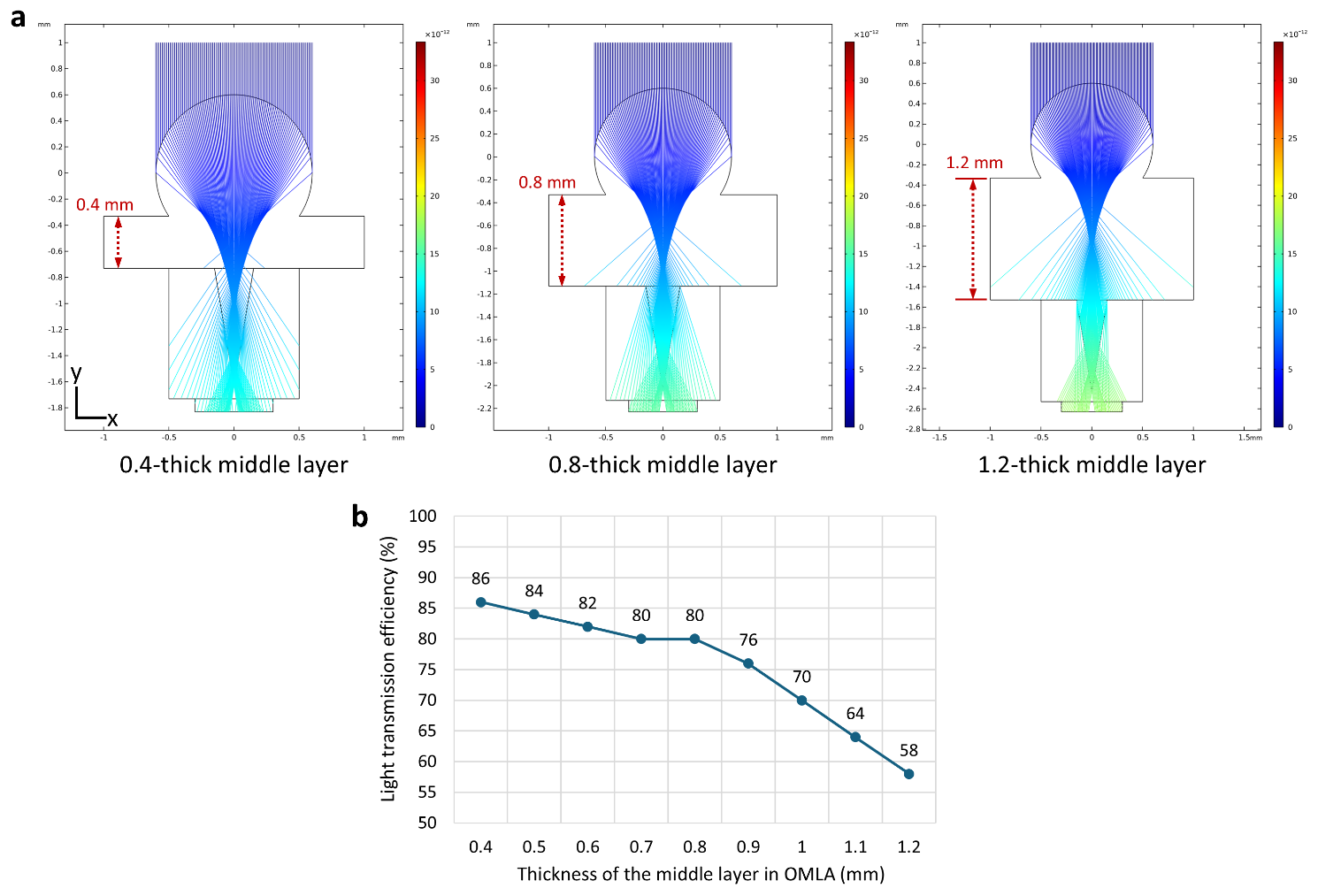  **Fig. S5** Ray tracing Simulation results with different thicknesses of a middle layer, (a) Plotting results of ray tracing with different thicknesses, (b) Change of light transmission efficiency by different thicknesses. |
| --- |

**References**

I. Bodurov, I. Vlaeva, G. Exner, Y. Uzunova, S. Russev, B. Pilicheva, A. Viraneva, T. Yovcheva, Ts. Grancharova, S. Sotirov, M. Marudova, J. Phys. Conf. Ser. 682, 012026 (2016). https://doi.org/10.1088/1742-6596/682/1/012026

A. Evan, N.G. Amaris, J. Julie, K. Indermeet, H. Iltefat, W.L. Henry, J.J. Jared, J. Am. Acad. Dermatol. 84, 1219 (2021). https://doi.org/10.1016/j.jaad.2021.02.048

T. Lister, P.A. Wright, P.H. Chappell, J. Biomed. Opt. 17, 0909011 (2012). https://doi.org/10.1117/1.jbo.17.9.090901

P. Makvandi, M. Kirkby, A.R.J. Hutton, M. Shabani, C.K.Y. You, Z. Baghbantaraghdari, R. Jamaledin, M. Carlotti, B. Mazzolai, V. Mattoli, R.F. Donnelly, Nano-Micro Lett. 13, 93 (2021). https://doi.org/10.1007/s40820-021-00611-9

F. Mikołajczyk, S. Kuberski, IOSR J. Polym. Text. Eng. 6, 14 (2019). https://doi.org/10.9790/019X-06031422

J. Park, K. Shobayashi, B. Kim, Micromachines 15, 725 (2024). https://doi.org/10.3390/mi15060725

S. Prahl, Monte Carlo Light Scattering Programs., http://omlc.org/software/mc/. Accessed 15 December 2024.

J.H. Pyne, E. Barr, E. Myint, S.P. Clark, M. David, R. Na, Clin. Exp. Dermatol. 43, 3 (2018). https://doi.org/10.1111/ced.13222

P. Vijitha, M. Manojit, IEEE Rev. Biomed. Eng. 10, 122 (2017). https://doi.org/10.1109/RBME.2017.2739801
